# Anæsthetics in Diseases of the Kidneys—Danger

**Published:** 1884-02

**Authors:** 


### Anæsthetics in Diseases of the Kidneys—Danger.

Dr. Laurence Turnbull dwells upon the great importance of attention to the condition of the kidneys and examination of the urine when an anæsthetic is to be administered. Many deaths, unaccountable otherwise, are due to this cause. In diseases of the kidneys, the blood being loaded with urea, anæsthetics almost invariably produce coma and death. He enumerates a considerable number of deaths from ether and hydrobromic ether, but very few from chloroform. Norris has reported two cases of death supervening unexpectedly from sulphuric ether after operations for cataract. Both recovered consciousness but died comatose, one in a few hours, the other after 18 days; no organic lesion was found post mortem except Bright's disease. Cases have also been reported by Emmet, Hunt and Montgomery, verified by post mortem examination. The kidneys are the active agents in eliminating ether from the blood, and if they are unable to perform this office, and if the skin is cold, moist and inactive, death will supervene by accumulation of mucus in the lungs, or congestion of the brain, in true Bright's disease of the kidneys.—*Med. and Surg. Rep.*

